# The Clinical Significance of Circulating Lymphocytes Morphology in Diffuse Large B-Cell Lymphoma As Determined by a Novel, Highly Sensitive Microscopy

**DOI:** 10.3390/cancers15235611

**Published:** 2023-11-28

**Authors:** Gil Fridberg, Galit Horn, Anat Globerson Levin, Dan Benisty, Sigi Kay, Chen Glait-Santar, Chava Perry, Ron Ram, Irit Avivi, Ben-Zion Katz

**Affiliations:** 1 The Hematology Division, Tel Aviv Sourasky Medical Center, Tel Aviv 6423906, Israel; gilfr@tlvmc.gov.il (G.F.);; 2 Immunology and Advanced CAR-T Therapy Laboratory, Tel Aviv Sourasky Medical Center, Dotan Center for Advanced Therapies, Tel Aviv Sourasky Medical Center, Tel Aviv University, Tel Aviv 6423906, Israel; 3 The Hematology Division, Tel Aviv Sourasky Medical Center, Faculty of Medicine, Tel Aviv University, Tel Aviv 6423906, Israel

**Keywords:** CAR T, lymphoma, morphology

## Abstract

**Simple Summary:**

Chimeric Antigen Receptor T-cell (CAR T) therapy has become the preferable therapeutic approach in patients with relapsed/refractory diffuse large B-cell lymphoma (DLBCL). Detection of CAR Ts in peripheral blood smear (PBS) is challenging due to the low sensitivity of current morphological technologies. We provide herein a morphological analysis of CAR Ts during the production process and in consecutive PBS obtained from DLBCL patients undergoing CAR T therapy, employing a novel, highly sensitive microscopy platform. We found that activated morphology was attributed predominantly to transduced cells following engagement with target cells. The average number of day 5 CAR Ts, and their sustained presence, were significantly higher in patients obtaining complete response. Also, a high number of CAR Ts was associated with longer cytokine storm release syndrome. These data indicate that CAR T morphological surveillance in PB might serve as a simple, fast and inexpensive method to provide clinically relevant insights.

**Abstract:**

Chimeric Antigen Receptor T-cell (CAR T) therapy has become the preferable treatment in relapsed/refractory diffuse large B-cell lymphomas (DLBCL) patients. Detection of CAR Ts in peripheral blood smear (PBS) is challenging due to insufficient data regarding their morphology and low sensitivity. The morphological evolution of CAR Ts along their production process, and in patients, was established by Full-Field Morphology (FFM), a novel digital microscopy approach that provides highly sensitive PBS analysis. At day 8 of production, 42.7 ± 10.8% of the CAR T transduced cells exhibited activated morphology compared with 9.3 ± 3.8% in untransduced cells. Moreover, engagement of transduced CAR Ts with target cells resulted in further morphological transformation into activated morphology (83 ± 5.6% of the cells). In patients, the average number of day 5 CAR Ts, and their sustained presence, were significantly higher in patients obtaining complete response. A high number of activated morphology CAR Ts at day 14 was associated with prolonged cytokine release storm. Overall, CAR Ts exhibited heterogeneous morphology, with the activated morphology attributed predominantly to transduced cells following engagement with target cells. Post-transfusion CAR T detection was associated with increased complete responses. FFM CAR T surveillance in PBS may serve as a simple inexpensive method to provide clinically relevant insights into this treatment modality.

## 1. Introduction

Chimeric Antigen Receptor T-cell (CAR T) therapy has become the preferable therapeutic approach for patients with relapsed and refractory diffuse large B-cell non-Hodgkin’s lymphoma (R/R DLBCL) [1,2,3]. Tisagenlecleucel [4] (Tisa-cel) and Axicabtagene Ciloleucel [5] (Axi-cel) are two FDA-approved CAR T therapies for the treatment of R/R DLBCL, providing durable responses in 30–40% of patients [6,7,8]. Consequential adverse effects include the cytokine release syndrome (CRS) reported in up to 93% of patients, with 13% to 22% of grade 3 or higher, and immune effector cell-associated neurotoxicity syndrome (ICANS), observed in up to 67% of patients, with 12% to 28% of grade 3 or higher [6,7,9].

Efforts to predict response to treatment and risk factors for the development of clinically significant adverse events focused on patient- and lymphoma-related characteristics [10] and evaluation of CAR T expansion post-transfusion [3,10,11,12,13]. Currently, there are no specific recommendations or commercial assays for routine CAR T measurements following transfusion, whereas polymerase chain reaction (PCR) or flow cytometry (FC) analysis are used in clinical studies for quantitative measurements of CAR T expansion and persistence [3,9,14,15]. Moreover, these modalities do not provide information about the cells’ functionality. In DLBCL, prognostic models based on CBC-derived lymphocyte counts have been suggested [16], including the use of lymphocyte counts kinetics to predict the outcome following CAR T treatment [17]. However, so far, no such models employing peripheral blood smear (PBS) analysis have been proposed. While morphological detection of circulating CAR Ts has been previously reported [17,18], the significance of such observations remains elusive.

Detection of CAR Ts in PBS is challenging due to insufficient data regarding their morphology prior to transfusion and low sensitivity of currently available morphological tools that enable the analysis of only a few snapshots of WBC, hence precluding a reliable analysis, especially in leukopenic patients. Scopio Labs Full-Field Morphology (FFM) is a novel digital microscopy platform that provides high-resolution images combined with a wide field of view that incorporates artificial intelligence classification capabilities [19]. The full-field/high-resolution combination enables the detection and classification of rare cells in PBS derived from leukopenic samples that are common during the first days following CAR T administration. In this study, we first characterized the morphology of CAR T cells in the products, prior to administration to patients. Next, the significance of the various CAR T morphologies was evaluated along the production process. Finally, we investigated the ability of the high-resolution/full-field FFM platform to detect and define CAR T morphology in PBS following transfusion, analyze CAR T kinetics and dynamics, and predict response to treatment and the development of treatment-related adverse events (particularly CRS) in R/R DLBCL patients treated with CAR T.

## 2. Materials and Methods

### 2.1. In Vitro Generation and Activation of CAR T

Peripheral blood mononuclear cells (PBMC) from three independent healthy donors were subjected to CAR T production process as detailed herein: T-cell transduction: retroviral transduction of T cells was performed as previously described [20,21]. After transduction, cells were cultured in the presence of 350 IU/mL IL^−2^. Target engagement of CAR T cells: CAR-T cells were co-cultured with target cells (Raji cell line, kindly provided by Tova Wax, Weizmann Institute of Science, Rehovot, Israel) expressing CD19 against which the CAR is directed. The effector:target ratio was 2:1. The cells were co-cultured for 16 h in RPMI and a concentration of 1 × 10^6^ lymphocytes/mL. The cells were then collected, stained against either CD19 (to detect target cells) or CAR and sorted accordingly. At various stages of the production process, cells were harvested, applied to glass slides (SP-Slides, Sysmex, Lincolnshire, IL, USA) and stained by the May Grunwald/Giemsa Staining protocol described below. The slides were scanned by a Scopio Labs X100 scanner (Scopio Labs, Tel Aviv, Israel) and subjected to morphological analysis.

### 2.2. Flow Cytometry Analysis and Sorting

Anti hCD3-APC (300439), anti hCD4-FITC (300506), anti hCD8-PB (301023) and streptavidin-APC (405207) were all purchased from BioLegend (London, UK). Rabbit Anti-CAR antibody was prepared in-house, cleaned and biotinylated. Cell surface markers, as well as a percentage of lymphocyte transduction, were analyzed by flow cytometry. Cells were incubated on ice for 30 min with the appropriate antibody. For CAR detection, a 2-step staining protocol was used. First, a 30 min incubation period with the biotinylated anti-CAR antibody was followed by 30 min incubation with streptavidin-APC. Staining and cell washes were carried out with 0.1 mL and 2 mL of FACS buffer, respectively (2% FCS, 0.05% sodium azid, 2 mM of EDTA in PBS). Cells were then analyzed by a FACSCantoII flow cytometer (Becton Dickinson, San Jose, CA, USA). Data analysis was carried out using FCS Express software. For sorting, cells were labeled with the designated antibodies, as described above, and were sorted with FACSAria III (Becton Dickinson, San Jose, CA, USA).

### 2.3. Preparation of Slides from CAR T Products

Attempts to prepare slides directly from the CAR T products, even when diluted into several types of media, such as phosphate-buffered saline, phosphate-buffered saline with 5% bovine serum albumin, RPMI tissue culture medium, without or with fetal calf serum, have all failed to provide high-quality samples adequate for morphological analysis. Hence, leftovers of CAR T transfusion bags (*n* = 3) were mixed with normal PB in a 1:3 ratio, aiming to preserve CAR T morphology in their natural environment, in a similar manner to a previous report [17]. Slides were then prepared from the CAR T/PB mixture.

### 2.4. Patient Cohort and Clinical Analysis

Twenty-six consecutive patients with R/R DLBCL who received either Axi-cel or Tisa-cel from October 2019 through October 2020 in the Tel Aviv Sourasky Medical Center (TASMC) were included (Table 1). Patients underwent a PET-CT scan one month post-CAR T infusion, and the response was defined according to the Lugano criteria [22]. CRS and ICANS grading were determined according to the American Society for Transplantation and Cellular Medicine (ASTCT) consensus [23]. Patient- and lymphoma-related characteristics at diagnosis and at CAR T administration, adverse events following CAR T transfusion and response to therapy were collected from patients’ medical records. Of note, none of the patients had a viral infection within the first 21 days post-CAR-T cell therapy. The study was approved by the local institutional review boards (IRB) according to the Declaration of the Helsinki Accord.

### 2.5. Peripheral Blood Smears’ Preparation and Staining

Peripheral blood samples were collected into a spray-dried K3 EDTA 3.6 mL vacuum tube (Greiner, Kremsmunster, Austria). PBSs were prepared, within four hours at room temperature, by a Beckman Coulter Slide Maker Stainer (Brea, CA, USA) on glass slides (SP-Slides, Sysmex, Lincolnshire, IL, USA). Staining protocol: Methanol (absolute, Bio-Lab Ltd., Jerusalem, Israel): 4 min 1 s; May Grunwald Stain (TruColor, Beckman Coulter, Brea, CA, USA): 6 min 22 s; Water (double-distilled, Terion, Cary, NC, USA): 3 min; Giemsa Stain (TruColor, Beckman Coulter, Brea, CA, USA): 9 min; Water (double-distilled, Terion, Cary, NC): 1 min; Air-dry: 6 min. All slides were covered with Süsse Cover Glasses, by using EUKITT^®^ Classic Mounting Medium Glue.

### 2.6. Morphological Analysis of PBS

Scopio Labs FFM locates the optimal analysis area for each PBS, including the monolayer area and the feathered edge. On average, the monolayer part of the scan was 2.21 (range 1.22–4.45) cm^2^, equivalent to 1000 high-power fields (100× magnification). Then, Scopio Labs FFM performs as described [19]. Alternatively, PBSs were analyzed by the CellaVision DM1200 system (Lund, Sweden), according to the manufacturer’s instructions in a DSS mode.

### 2.7. Flow Cytometry

Clinical flow cytometry was performed on PBSs on day 7 after CAR-Tc infusion. The cells were stained with the combination of 10 μg/mL of FITC-labeled human CD19 (20-291) protein Fc-Tag (Acrobiosystems Inc., Newark, DE, USA) and CD3-APCH7 (BD Biosciences, San Diego, CA, USA). Samples were acquired by FACS Canto II and analyzed by DIVA software (BD Biosciences, San Diego, CA, USA).

### 2.8. Laboratory Data

Laboratory results, including complete blood counts (CBC) and differential, CRP, LDH and ferritin were obtained from patients’ medical records.

### 2.9. Statistics

Statistical analyses were performed with SPSS software (Version 27, IBM Corp., Armonk, NY, USA). A two-tailed *p* value < 0.05 was considered statistically significant. We used the Mann–Whitney test to compare continuous variables between categories. Spearman’s rank correlation coefficient was used to quantify the association between continuous variables. Friedman and Wilcoxon tests were used to compare results between periods and two types of platforms (FFM and CellaVision).

## 3. Results

### 3.1. The Diverse CAR T-Cell Morphology

**Figure 1:**
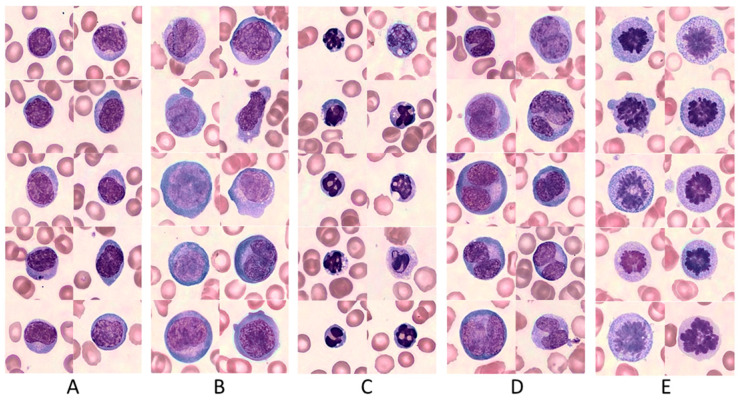
Pre-infusion CAR T morphology. (**A**) Quiescent lymphocytes; (**B**) Activated morphology lymphocytes; (**C**) Apoptotic lymphocytes; (**D**) Multi-nucleated lymphocytes; (**E**) Mitotic lymphocytes. The cells represent both Tisagenlecleucel and Axicabtagene Ciloleucel products. Images were taken at 100× magnification.

**Figure 2:**
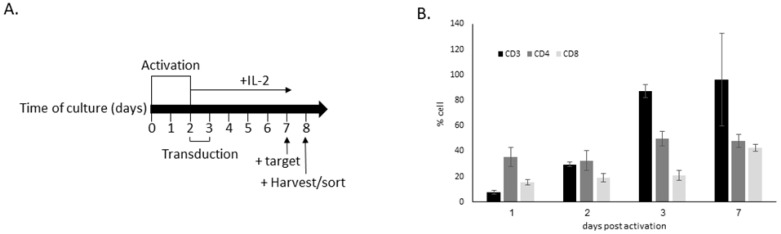
The production process of CAR T, and lymphocytes characterization. (**A**) CAR T production and activation timeline. Time points of activation of the cells with anti-CD3/CD28, CAR transduction, incubation with IL-2, addition of target cells and sorting/harvesting of the experiments are indicated. The average transduction percentage was 32.56% on day 7 (±2.98%, *n* = 3) (**B**) Percentage of cells expressing CD3, CD4 and CD8 at indicated days post activation, analyzed via flow cytometry (αhCD3-APC, αhCD4-FITC, αhCD8-PB). The results are the average ± S.D. of three productions.

**Figure 3:**
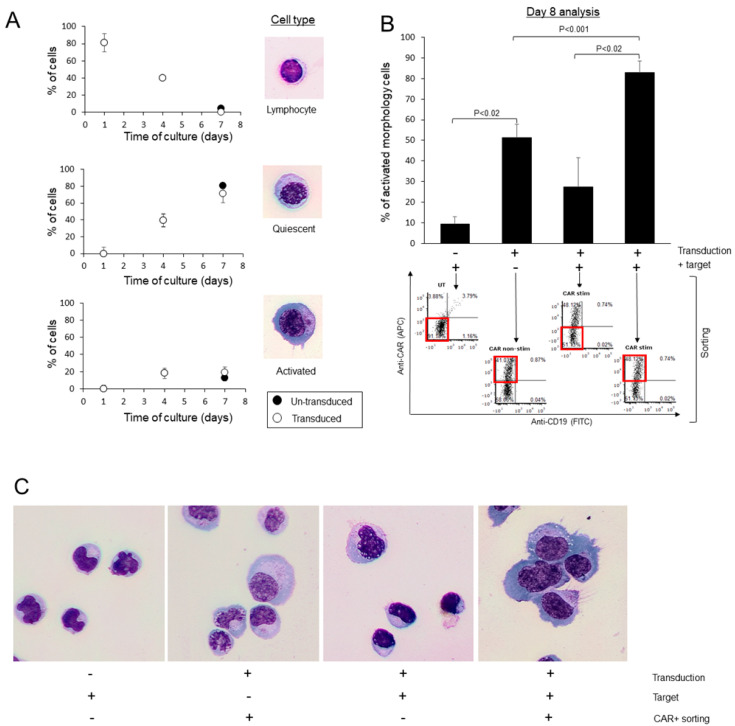
CAR-T cells exhibit activated morphology following specific target encounter in vitro. (**A**) percentage of cells exhibiting the lymphocytes (top), quiescent (middle) and activated (bottom) morphology at indicated days post activation of transduced (white circles) or un-transduced cultures (black circles). The results are the average ± S.D. of three productions. (**B**) percentage of cells exhibiting activated morphology following co-culture with CD19+ tumor cells. On day 7, transduced or un-transduced lymphocytes were co-culture in a 2:1 effector:target ratio with the CD19 expressing Raji cell line for 16 h. Cultures were then sorted, and all CD19 negative cells, which were sorted into CAR-positive or CAR-negative cells, were collected. Sorted cells (red squares) were fixed and stained and the morphology was determined via microscopic examination. The results are the average ± S.D. of three productions. *p* values are presented, with *p* < 0.05 considered significant. (**C**) Representative images of cells on day 8 of the experiment, with each treatment designated at the bottom.

**Figure 4:**
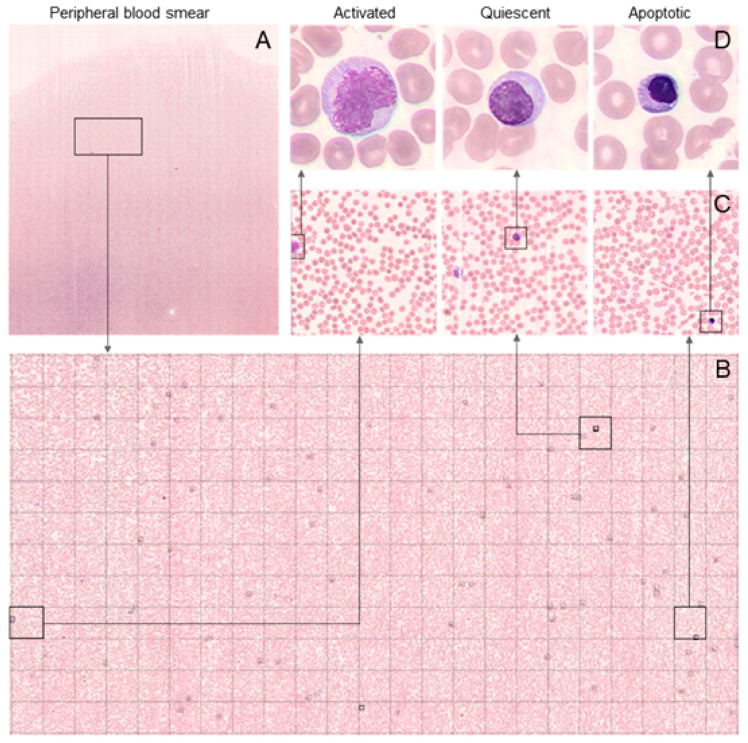
Acquisition and classification of CAR T in PBS by FFM. A portion of the scanned slide is shown (**A**), where each square represents a 100× magnification FOV (**B**). WBCs were detected (**C**) and classified manually by a morphology expert according to their morphology. Representative examples for activated (left), quiescent (middle), and apoptotic (right) morphology are shown (**D**).

Since the morphology of CAR T prior to transfusion was not yet established, we first created a basal CAR T morphological library. A total of 5367 cells (average of 1789 per smear, range 59–3411), obtained from three product smears (2 Tisa-cel, 1 Axi-cel), were used to characterize basal CAR T morphology. Five morphologically distinct subpopulations were identified: (1) Quiescent morphology lymphocytes, small lymphocytes with slightly enhanced cytoplasm, and basophilic cytoplasm (Figure 1A); (2) Activated morphology CAR T, large cells with abundant basophilic cytoplasm and reticulated nucleus, with or without nucleoli (characteristics which are typical for reactive lymphocytes) (Figure 1B); (3) Apoptotic cells, small lymphocytes with pycnotic nuclei, often with vacuoles (Figure 1C); (4) Multinucleated lymphocytes (Figure 1D); and (5) mitotic lymphocytes, referring to cells in mitosis, mostly in anaphase (Figure 1E). Supplementary Figure S1 depicts the differences between normal, quiescent and activated lymphocytes’ morphology as defined in this study. Similar morphological types were identified both in the Tisa-cel and Axi-cel products.

### 3.2. Characterization of CAR T-Cells’ Morphology during Their Production Process

In order to validate morphology as a reliable tool to detect, measure and follow CAR T in PBS, we characterized the morphology of the cells during the various stages of the production process (Figure 2A). On day 1, the cultures contained 81.3 ± 5.0% normal morphology lymphocytes, and the rest were predominantly monocytes (Figure 3A and Figure S2A). Of these cells, only 7.7 ± 5.1% were CD3+ T cells with a CD4/CD8 ratio of 2.27 (Figure 2B). The cells were then subjected to CD3/CD28 activation (days 1–2) and transduction (days 2–3). IL-2 was added to the cultures during days 2–7 (Figure 2A). Untransduced cells served as controls. On day 4, 40.0 ± 11.0% of the cells retained normal lymphocyte morphology, while 39.3 ± 7.4% of the cells were with quiescent morphology, and the rest predominantly activated morphology lymphocytes (Figure 3A). Note that 87.04 ± 5.0% of these cells were CD3+ T cells, with a CD4/CD8 ratio of 2.39 (Figure 2B).

On day 7, no lymphocytes with normal morphology were observed among the transduced cells, with 71.3 ± 7.0% of quiescent and 19.0 ± 1.7% of activated CAR T morphologies (Figure 3A). In the untransduced cultures of day 7, 80.3 ± 8.1% of quiescent and 13 ± 6.2% of activated CAR T morphologies, as well as 4.3 ± 2.3% of lymphocytes with normal morphology, were observed (Figure 3A and Figure S2B). In the transduced cultures, 96.38 ± 1.56% were CD3+ T cells, with a 1.13 CD4/CD8 ratio (Figure 2B). Low percentages of apoptotic cells (1.2–2.6%) appeared in the different cultures at days 0–7, while mitotic cells (0.6–7%) were observed in cultures of days 4 and 7, and only solitary binucleated cells were observed (not shown). The morphological diversity observed at the end of our production process was similar to that found in the commercial products (Figure 1).

### 3.3. Assessment of CAR T Morphological Significance

At day 7, transduced or untransduced cultures were incubated for 24 h with a CD19+ Raji B-cell line that served as target cells. At day 8, the various cells were sorted for CAR+ or CAR- expression. As shown in Figure 3B, only 9.3 ± 3.8% of the untransduced cells incubated with target cells exhibited activated morphology, while most of the cells were small, with relatively dense nuclei (Figure 3C). Of note, 42.7 ± 10.8% of the CAR+ cells sorted from the transduced population that was not exposed to target cells had an activated morphology (Figure 3B,C); 27.3 ± 14.3% of the CAR- cells sorted from the transduced population that was exposed to target cells had an activated morphology (Figure 3B,C). However, 83 ± 5.6% CAR+ cells sorted from the transduced population that was exposed to target cells had an activated morphology, significantly higher than all the other groups (Figure 3B,C). Interestingly, in the sorted CAR+ cell population that was incubated with target cells, conjugates between activated morphology CAR T and apoptotic cells were observed, with granules in the CAR T concentrating within the conjugation area (Supplementary Figure S2C, inserts).

### 3.4. Patients’ Characteristics

Twenty-six R/R DLBCL patients (12 males), were included in the study. The median age at CAR T transfusion was 71 years (20–84). Seventeen patients entered CAR T with stable/progressive disease. Twenty-one (80.7%) patients received Tisa-cel, and five (19.2%) received Axi-cel. Eighteen patients (69.2%) developed CRS (3 (11.5%) grade ≥ 3), and seven (36.9%) developed ICANS (2 (7.69%) grade ≥ 3). The median durations to CRS and ICANS from infusion were 3 and 6 days, respectively. PET scans performed at one month post-CAR T infusion revealed that 13 patients (50%) attained a complete metabolic response, 5 (19.2%) obtained a partial response and 8 (30.7%) had stable or progressive disease (patients’ characteristics are presented in Table 1).

### 3.5. Evaluation of CAR T Morphology in PBS, Following Infusion

The capacity of FFM analysis to retrieve a high number of cells even from leukopenic samples is demonstrated in Figure 4. First, a large area of the PBS was scanned containing an average of 1000 Fields of View (FOVs) (Figure 4A). Then, individual WBCs were detected throughout the scan (Figure 4B). Each square represents a 100× magnification single FOV (Figure 4B,C). The identification of cells in each PBS was determined manually (Figure 4D). In order to compare the performance of current digital technologies, Scopio Labs FFM vs. the CellaVision DM1200 platforms, 77 blood smears obtained from 18 patients were examined by both technologies. Overall, 24,247 WBCs were detected by the Scopio Labs FFM platform (average 303 cells per PBS, range 0–2295), compared with 4335 detected by the CellaVision DM1200 system (average 54.4 cells per PBS, range 0–146). During the first four days post-infusion, when the patients were leukopenic (<1 × 10^3^ WBC/mL), the CellaVision DM1200 system could hardly detect any CAR T in PBSs, with a median number of two cells only (IQR 1–2), compared with five (IQR 2–9) when using the Scopio Labs FFM platform. Scopio Labs FFM provided a higher number of CAR T in 74% (*n* = 57) of the PBSs, an identical number of CAR T in 16.8% (*n* = 13), and a lower number of CAR T in 9% (*n* = 7) of the PBSs. Overall, the difference between the two methods was significant, with a superior detection rate of the Scopio Labs FFM technology (median difference 2, IQR 0–6, *p* value < 0.001).

One hundred sixty-six consecutive PBSs, collected from 26 patients on days 1–15 following CAR T transfusion, were analyzed by the FFM platform. Automated detection and classification of CAR T into the five described categories were performed, and the results were validated manually by two independent hematopathology experts. Multinucleated and mitotic cells were scarce and, therefore, withdrawn from the final analysis. The average total WBC number/PBS was 453.7 ± 280.8 (range 262–1270), relatively stable along days 1–15 following administration. The average number of total CAR T/PBS was 11.0 ± 5.0 (range 2.5–19.4) and remained relatively stable throughout days 2–15. Overall, CAR T accounted for a very small proportion of the total WBC in the PBS, with an average percentage of 2.8 ± 1.4% (range 0.63–5.7%). None of the specific CAR T morphologies appeared in these patients 1–3 days prior to the administration of the cells.

### 3.6. Association between CAR T in PBS and Patients’ Outcomes

**Figure 5:**
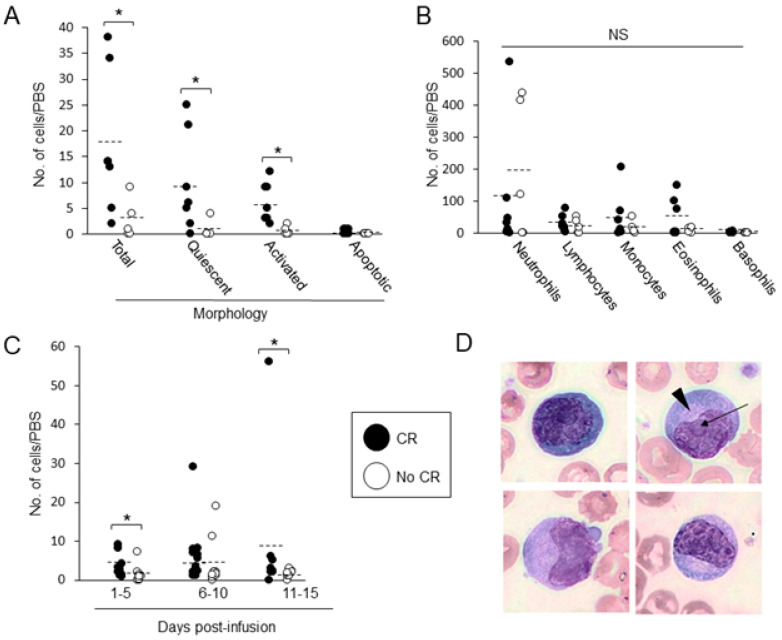
Correlations between CAR T in PBS and response. (**A**) Average ± S.D. of lymphocyte morphological subtypes/day 5 PBS was determined in patients who achieved (white bars) or did not (black bars) achieve complete response (CR). * = *p* < 0.03. (**B**) The average ± S.D. of basophils, eosinophils, monocytes, lymphocytes and neutrophils/day 5 PBS was determined in patients who achieved or did not achieve CR. NS = non-significant. (**C**) The average ± S.D. of activated morphology CAR T was in 5 days intervals post transfusion in patients who achieved or did not achieve CR. * = *p* < 0.03. (**D**) Typical D5 activated morphology CAR T. Arrow indicates nucleoli, arrowhead points to white peri-nuclear hallow. Images were taken at 100× magnification.

The average number of total CAR Ts, measured on D5 post-transfusion, was significantly (*p* = 0.018) higher in patients who obtained a complete response at day 30 post-CAR T, reaching 17.1 ± 13.7 cells/PBS (range 2–38) vs. 2.8 ± 3.4 cells/PBS (range 0–9) for patients who did not reach CR (Figure 5A). D5 quiescent cells and activated CAR Ts were particularly higher in patients attaining CR: 9.7 ± 9.6 cells/PBS (range 0–25) vs. 6.1 ± 3.8 cells/PBS (range 2–12), *p* = 0.023; and 0.8 ± 1.8 cells/PBS (range 0–4) vs. 0.6 ± 0.9 cells/PBS (range 0–2), *p* = 0.005, respectively (Figure 5A). No such correlation was found with other leukocyte types in the PBSs (Figure 5B). In line with that, the presence of CAR Ts in PB, assessed by measuring the average number of activated CAR Ts on D1–5 and D11–15, was also associated with increased CR rates; average numbers of activated morphology CAR Ts were 3.4 ± 2.9 cells/PBS (range 0.75–9, days 1–5), and 9.5 ± 18.9 cells/PBS (range 0–56, days 11–15) in patients with CR, vs. 2.2 ± 0.9 cells/PBS (range 0–7, days 1–5) and 1.5 ± 0.9 cells/PBS (range 0–3, days 11–15) in patients who did not reach CR (*p* = 0.03 and *p* = 0.04, respectively) (Figure 5C). The activated morphology CAR Ts, measured on day 5, were large cells with abundant basophilic cytoplasm and reticulated nucleus, with (Figure 5D, arrow) or without nucleoli, and evident perinuclear hallow representing developed Golgi in some of the cells (Figure 5D, arrowhead). As expected, disease burden was also associated with the outcome; patients entering CAR T therapy with responsive disease (*n* = 17) had a greater chance of achieving CR following transfusion compared with their nonresponding counterparts (*n* = 9) (*p* = 0.015).

### 3.7. Association between CAR T in PBS and Adverse Events

**Figure 6:**
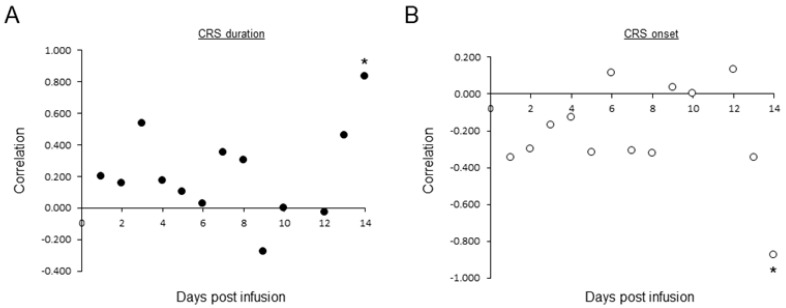
Correlation between activated morphology CAR T and CRS. In total, 166 consecutive PBSs of 26 patients were prepared and scanned on days 1–14 post CAR Ts transfusion. Activated morphology CAR T were classified manually by morphology experts. CRS severity grading was determined according to the American Society for Transplantation and Cellular Medicine (ASTCT) consensus. Correlations between CRS onset (**A**, black circles) or CRS duration (**B**, white circles) and average number of activated morphology CAR T/PBS were determined during days 1–14. * = *p* < 0.03 (left).

We examined the association between CAR T in PBS and the occurrence of adverse events. There were no statistically significant associations between specific morphological subtypes and CRS development. However, continuous detection of activated morphology CAR Ts through days 1 to 14 tended to be associated with a longer duration of CRS (Figure 6A). Large numbers of activated morphology CAR Ts in day 14 PBSs were significantly correlated with long CRS (Figure 6A) and negatively correlated with early CRS onset (Figure 6B). ICANS development was scarce in our cohort, not enabling an analysis.

## 4. Discussion

Currently, CAR T in the PB is measured either by transgene level with PCR [13,14,24] or by FC [9,15,24]. FC detection depends on surface CAR expression [25]; thus, the CAR T amount might be an underestimation, and PCR levels are highly variable between products and patients [22]. Both methods are expensive, require elaborate laboratory protocols, and do not provide any information regarding the functionality of CAR+ cells. The cellular kinetics of CAR Ts evaluated in patients treated with CAR Ts for R/R acute lymphoblastic leukemia revealed a complex concordance relationship between PCR and FC measurements; in some cases, cellular expansion was observed by qPCR, but FC indicated that this was not a result of functional CAR Ts [26], suggesting that both methods are not fully accurate. Moreover, both these methodologies provide only quantitative data concerning the presence of the CAR, either at the DNA or protein levels, but do not provide any information regarding the functionality of the effector cells. We found a weak correlation (*n* = 10, r = 0.67, *p* = 0.034) between the time to peak of CAR Ts in the PBSs to the CAR+ cells number as measured by flow cytometry on day 7 post-transfusion. A recent review article indicated that current methodologies of CAR T cells’ quantification are extremely heterogeneous and difficult to compare and standardize [27].

Morphological analysis of PBS is routinely performed for monitoring patients with hematological malignancies. PBS morphological analysis enables us to distinguish between cell subtypes in heterogenic pools, differentiate between viable and nonviable cells, and is compatible with a high-throughput routine workflow, being a simple, quick and inexpensive technique. We demonstrate herein the feasibility of CAR T detection in PBS, utilizing a novel, highly sensitive FFM approach. As we show in this study, CAR Ts comprised a very small proportion of total leukocytes in PBS, sometimes too low to enable their detection by currently available digital morphology technologies. Moreover, current quantitative morphology (e.g., CellaVision) cannot be performed, specifically in leukopenic samples, since an average of only 54 cells/PBS are detected, less than the minimum of 100 cells required for differential analysis [28]. The FFM approach retrieves significantly higher WBC numbers from each PBS, even in leukopenic patients, a common condition during the first few days following CAR T administration. Thus, continuous quantification of PB CAR T cells can be performed in most samples with the highly sensitive Scopio Labs technology and correlated with clinical outcomes.

In order to elucidate the significance of CAR T morphology, we first analyzed in vitro the morphological evolution of the cells along the production process. During days 1–7 of the production process, the lymphocytes’ morphology is transformed into cells with basophilic cytoplasm with reticulated nuclei, accompanied by an increase in CD3+ proportion of the total lymphocytes, and an increase in the CD3+/CD8+ proportions of the lymphocytes population. This indicates that activation of the T cells with CD3/CD28 engagement and continuous exposure to IL-2 is only sufficient to generate basophilic cytoplasm and loosen the chromatin, but CAR transduction and, moreover, target engagement, are required to achieve full activated morphology. Interestingly, in CAR T incubated with target cell, conjugates between the immune and apoptotic cells can be observed, with granules concentrated in the engagement area, consistent with the degranulation required to induce apoptosis in CAR T targets [29].

As we show here, specific morphological forms can be identified in the CAR T end products prior to their infusion, from quiescent to activated to apoptotic cells, including cells in mitosis. The quiescent lymphocytes differ from normal lymphocytes by the slightly enhanced basophilic cytoplasm, apparently due to exposure to IL-2 during the production process. Most of the mitotic forms that we identified were in the anaphase stage of mitosis, or cells following mitosis without cellular partition, and their presence indicates that the products contain proliferating cells while being introduced into the patient’s circulation. These mitotic forms were only observed in the products but not in patients’ PBS throughout days 1–14 following infusion. Solitary binucleated cells appeared in the CAR T products, and they may be the outcome of abnormal cell partition during the mitosis of these highly proliferative cultures. The significance of the morphological heterogeneity of the products remains to be established. Excessive CAR T differentiation (caused by mechanisms pushing the cell to certain differentiation pathways) leads to exhaustion, senescence as well as a reduction in expansion and persistence, resulting in dysfunctional phenotype and vice versa [30,31,32]. As shown here, a portion of the circulating CAR T that exhibited activated morphology through days 2–14 post-infusion, is characterized by abundant basophilic cytoplasm, immature nuclei and well-developed Golgi, all potentially compatible with the potential for extensive secretion of various cytokines [13]. As we found, their presence on day 14 post-transfusion indeed correlates with prolonged CRS.

We found that the total presence of CAR T in day 5 PBS correlated with the clinical outcome of the treatment, but the presence of the activated form throughout days 1–14 was also important. Previous studies indicated that both products used in our cohort have an expansion peak of CAR T around days 7 to 14 [6,7,33,34,35]. In the pivotal JULIET study of Tisa-cel, similar expansion patterns, measured by PCR, were observed between those who responded and those who did not [7]. In contrast, in the BELINDA trial (Tisa-cel as 2nd-line treatment), the mean in vivo expansion was two times higher in patients who responded to treatment compared with those who did not. Moreover, patients who exhibited a higher CAR T expansion had a longer event-free survival [36]. In the pivotal ZUMA-1 study of Axi-cel, expansion peaked within 14 days after infusion and was associated with response to treatment [33]. Consistent trends were observed in the ZUMA-7 trial (Axi-cel as 2nd line) where CAR T cells peak during the first 28 days correlated with the response [33]. Real-world data of patients with stable or progressive disease, treated with either Tisa-cel or Axi-cel, showed that at day 7, CAR T levels (measured by FC) were predictive for both short and long outcomes [36]. According to our data, morphological detection of CAR Ts in PBS, predominantly in their activated morphology, was also found to predict the achievement of clinical response. As we show in this study, the activated morphology is typical of CAR+ cells, particularly following engagement with the malignant cells, and hence may indicate clinically productive cells.

We acknowledge several limitations. Being a pilot study employing novel technology, it included a low number of patients and, consequently, a limited number of samples. The majority of patients (21) were treated with Tisa-cel, and our study was not accompanied by comparative molecular studies. The morphology of activated CAR T cells resembles that of activated T cells that can be found in cases with viral infections. There are several factors that minimized assignment errors: 1. A library of morphological features was established from the CAR T product itself, reviewing a very large number of CAR Ts. 2. Morphological features were distinct, ensuring a reliable detection of CAR Ts vs. all other PB cells. 3. As expected, PBS obtained during the first days post-CAR T transfusion were leukodepleted, therefore presenting mostly CAR T prior to bone marrow recovery. 4. None of the patients who participated in the study were diagnosed with a viral infection. It should be noted that surveillance PCR tests for CMV were taken for all patients, as part of their routine management, and PCR tests for respiratory viral infections were taken from all patients if they developed a fever. 5. Our analysis failed to demonstrate any correlation between any other WBC subclasses and outcomes post-CAR T but with our defined CAR T only. Furthermore, our finding demonstrating a positive correlation between day 5 CAR T measurement and the achievement of CR corresponds with clinical trials based on molecular methods [6,7,34,35,36]. It is worth mentioning that previous studies, evaluating the cellular kinetics of Tisa-cel in B-cell ALL and chronic lymphocytic leukemia (CLL) transfused patients, showed that most circulating lymphocytes in the first days after infusion are CAR Ts [27]. Furthermore, the kinetics of atypical lymphocytes post-Tisa-cel (in pediatric ALL patients) had morphological changes that were distinct from other cell lineage lymphopoiesis or maturation, thus inferred to be CAR T [17].

## 5. Conclusions

We show in this study that CAR T cells exhibit heterogeneous morphology identified in PBS by the highly sensitive FFM morphological approach. The diverse morphology expresses a complex production process yielding various end products, with the activated cellular morphology associated with CAR+ expression and particularly following target cells’ engagement. Activated morphology CAR T in PBS, detected in specific days, was associated with outcome and therapy-related toxicities, emphasizing that CAR T monitoring requires quantitative, qualitative and repeated measurements. Considering the aforementioned limitations of FC and PCR together with the diversity in CAR T products, morphological measurement of CAR T dynamics might bring additional relevant information. Our findings, if confirmed in a larger cohort, could support the employment of morphological CAR T surveillance using FFM, being a simple, informative and inexpensive method to provide clinically relevant insights.

## Figures and Tables

**Table 1:** Baseline characteristics, outcome and adverse events of patients.

Characteristics at CAR T Transfusion			
Variable	All Patients	Patients’ Characteristics by Product	
		Tisagenlecleucel	Axicabtagene Ciloleucel
No. of patients	26	21	5
Disease type—no. (%)			
DLBCL	23 (88.5)	19 (90)	4 (80)
PMBCL	3 (11.5)	2 (7.7)	1 (20)
Age–Median (range) yr.	71 (20–84)	71 (20–84)	72 (27–74)
Gender (Male)—no. (%)	12 (46.1)	11 (52.3)	1 (20)
IPI Score—no. (%)			
0–2	5 (19.2)	5 (23.8)	0 (0)
3–4	21 (80.7)	16 (76.1)	5 (100)
Prior therapies—no. (%)			
1–2	17 (65.3)	15 (71.4)	2 (40)
≥3	9 (34.6)	6 (28.5)	3 (60)
ECOG status score of 1—no. (%)	11 (42.3)	10 (47.6)	1 (20)
LDH (U/L) before infusion, Median (range)	402.5 (215–3636)	401 (215–3636)	421 (372–553)
Response and adverse events following CAR T treatment			
Response at one month—no. (%)			
CR/PR	13 (50)/5 (19.2)	10 (47.6)/4 (19)	3 (60)/1 (20)
SD/PD	0 (0)/8 (30.7)	0 (0)/7 (33.3)	0 (0)/1 (20)
CRS—no. (%)			
Any grade	18 (69.2)	15 (71.4)	3 (60)
Grade 1–2	15 (57.6)	12 (57.1)	3 (100)
Grade ≥ 3	3 (11.5)	3 (14.2)	0 (0)
ICANS—no. (%)			
Any grade	7 (26.9)	5 (23.8)	2 (40)
Grade 1–2	5 (19.2)	2 (15.3)	2 (40)
Grade ≥ 3	2 (7.69)	3 (14.2)	0 (0)

The abbreviation CR denotes complete response, CRS: cytokine release syndrome, DLBCL: diffuse large B-cell lymphoma, ECOG: eastern cooperative oncology group, ICANS: immune effector cell-associated neurotoxicity syndrome, IPI: international prognostic index, PD: progressive disease, PMBCL: primary mediastinal B-cell lymphoma, PR: partial response, SD: stable disease.

## Data Availability

Data are contained within the article or Supplementary Materials.

## Supplementary Materials

The following supporting information can be downloaded at: https://www.mdpi.com/article/10.3390/cancers15235611/s1, Figure S1: Lymphocytes morphology; Figure S2: CAR T morphology during the production process.
