# Supplementary material for: The Clinical Significance of Circulating Lymphocytes Morphology in Diffuse Large B-Cell Lymphoma As Determined by a Novel, Highly Sensitive Microscopy
Source: Cancers (Basel). 2023 Nov 28;15(23):5611. doi: 10.3390/cancers15235611 (PMC10705094; doi:10.3390/cancers15235611)
Supplement: Supplementary file 1 [file cancers-15-05611-s001.zip › cancers-2716293-supplementary.pdf]

Supplementary materials

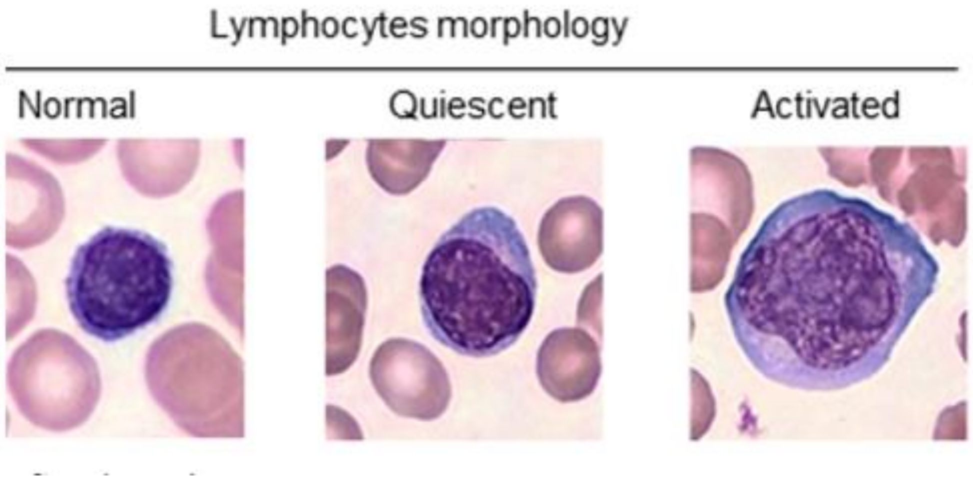

Figure S1. Lymphocytes morphology. Normal – normal morphology lymphocytes. Quiescent – A lymphocyte that passed the CAR T production process, but do not express the CAR. Activated – A lymphocyte that passed the CAR T production process, and mostly express CAR and engaged a target cell. Images were taken at 100 × magnification.

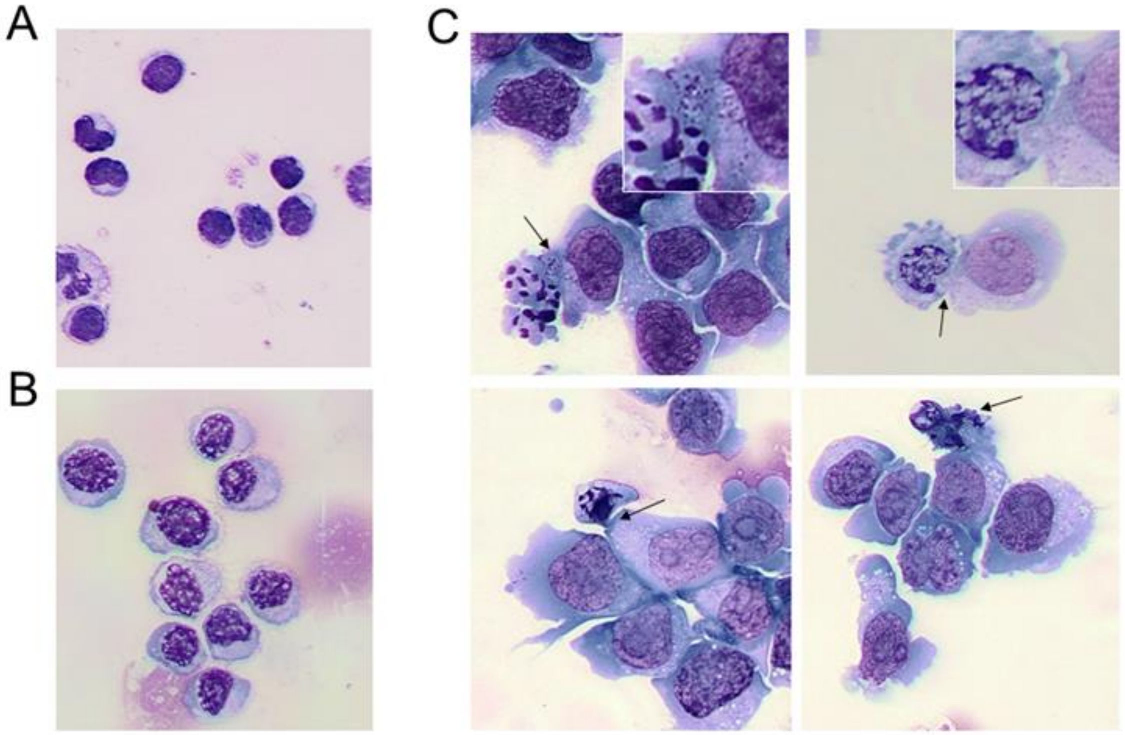

Figure S2. CAR T morphology during the production process. (**A**) Day 1 The cultures contained mostly normal morphology lymphocytes, and the rest were predominantly monocytes (**B**) Day 7. Un-transduced cultures. Most of the cells exhibited a quiescent morphology. (**C**) Day 8. Sorted CAR+ cells that incubated with target cells. Conjugates between activated morphology CAR T and apoptotic cells were observed (arrows), with granules in the CAR T concentrating within the conjugation area (top images, inserts). Images were taken at 40 × magnification.
